# Indents

**Published:** 1907-08-15

**Authors:** 


### INDENTS.

☞ **Brief Articles of Technical or Scientific Value will receive notice and comment. Contributions invited.**

**Prevent Pain in Filling Children's Teeth.** Where children come with large cavities in molars, but with no pulp exposure as yet, the thorough and often painful (painful if thorough) removal of decay may be avoided if the nitrate of silver treatment is used prior to inserting a filling; and thus our work is durable, while tediousness and pain are largely eliminated, and the child is our friend.—R. B. Tuller, *American Journal.*

**Therapeutical Notes.** (1) Ointment for neuralgia:

R—Mentholis......gr xij;
Cocainae......gr. iv;
Chloralis......gr. ij;
Petrolati......gr. lxxv. M.
Fiat unguentum.
Sig.—Apply to the painful part and cover with a gauze bandage, if the neuralgia is perorbital or hemicranial.

(2) Mouth-wash for diabetics:
Croftan, in the *Clinical Review*, recommends:

R—Beta-naphtholis......gr. v;
Sodii borati......ʒvj;
Aquae menth. pip......fʒvj;
Aquae destillatae......Oj. M.
Sig.—To be used as a mouth-wash.

Also for *bleeding gums* the following should be used:

R—Tinct. opii......fʒvj;

Potassae chloratis......

Sodii boratis......aa ʒijss;

Decocti althaeae radicis......Oj. M.

Sig.—To be applied to the gums.

(3) For constitutional treatment of alveolar abscess;

R—Iodii sulphatis ʒi;

Aquae ℥v;

Syrupus ℥j; M.

Sig.—One tablespoonful every hour.

The pain will subside slowly after three to four doses.—*N. Y. Medical Journal.*

**Electric Sleep.** Professor Leduc, of Nantes, has published the remarkable results he has obtained by means of his electric sleep. He gives this name to a condition comparable to chloroform narcosis, in which the subject lies without any powers of sensation or of voluntary motion, only certain reflex movements and the action of the heart and of the respiratory functions persisting. This condition is produced by the action on the brain of a certain form of electric current, and may be maintained for hours, and brought to an end instantly with the interruption of the current. The current used to produce the electric sleep is intermittent, of low tension and constant in direction; that is to say, it flows for a certain period. stops, and then flows again at perfectly regular intervals. It is generated by means of a source of continuous current and of a specially constructed form of interrupter. On applying it to the head, sensations of taste and of light are produced, and vertigo, depending to some extent on the manner in which the electrodes are applied, is caused. In order to inhibit motility and sensation the current should attain a voltage of about six, but if it is increased to ten volts respiration and heart action cease and death follows. Leduc has subjected himself to this action and has reported in a very interesting manner the sensations produced during a period of narcosis lasting twenty minutes. A remarkable fact is that when the flow of current is interrupted the subject awakes immediately without any of the after-effects that follow chloroform narcosis.—*Medical Record* (Paris letter).

**"Sticky-Wax" Casting.** Obtain several—say a dozen—lengths of glass tubing. Pieces about one foot long are convenient, but much longer pieces may be used. The tubing should have fairly thick walls. The bore may be of any diameter, though three-sixteenths to one-fourth inch gives convenient sticks of wax. The ends of the tubes should be ground, not melted smooth, as melting lessens the bore at the point of fusion.

Thoroughly clean the tubes and dry the inside by pushing pieces of cotton wool through them. The tubes must then be "lubricated" to prevent the wax from sticking. Do not lubricate with oil. Glycerine forms a very effective lubricant for this purpose, and is easily applied by saturating pieces of cotton wool with it and pushing them through the tubes.

Having the wax melted and the tubes "lubricated," fill each of the tubes as follows: Attach a piece of rubber tubing to one end of a glass tube. Take the other end of the rubber tubing in the mouth. Dip the free end of the glass tube into the melted wax and suck the wax up until the tube is full. Pinch the rubber tubing close to the glass tube, and the tube, full of wax, can then be lifted and laid in a horizontal position. Then release and remove the rubber tubing. The wax will not run out. Repeat the process until all the tubes are filled. When cool, the sticks of wax can readily be pushed out of the tubes. If a piece of wax should stick in a tube, it is either because the tube was not properly lubricated or because its bore was not uniform. I have cast sticks of wax over a yard long by his method. In melting the wax it is better not to use direct heat, but to put the wax in a tin and stand the tin in boiling water. Ordinary pink wax may be cast into sticks for "waxing up" in the same manner, but owing to the much greater fluidity of pink wax when melted, it is more difficult to manipulate.

The following is a formula for sticky wax: Pure white beeswax 4 oz., pure light yellow resin 7 oz., pure gum dammar 1 oz., dye q. s. to color. Powder the resin and gum dammar, and add little by little the melted beeswax.—*Dental Record.*

**Higher Ideals.** We take it as a matter of course that everything about the office should be clean. We demand clean floors, clean linen, clean instruments, a clean body outwardly, in order that we may not offend the esthetic in our patients, and why not demand of ourselves clean living? Would you not prefer, if your wife or daughter were in another city and required the services of a dentist other than yourself that they go to one whom you knew had both clean hands and a pure heart? Is it too much to ask, sinse railroads and other large corporations are demanding abstinence from alcoholic liquors during working hours and some of them total abstinence of their employes, that the men who have the care of the mouths of our wives and daughters, shall come to their work in the morning free from the odor of last night's dissipation, exhaling a mixture of bourbon, tobacco, welsh rarebit and limberger cheese? Is it too much to demand that the hand that holds the chisel and directs the maneuverings of an electric engine bur, in the most sensitive of human tissues be free from the unsteadiness that results from late hours, alcoholic stimulants and narcotics? Is it too much to demand that hand, eye, and brain shall be in the best possible condition for the trying situations that they have to meet?

Shall we guard the oral cavity from contamination by unclean hands and unsterile instruments, but leave the citadel of the heart open to befoulment by gross suggestion from one who is an habitue of the saloon and the brothel, even though they be palatial in their furnishings and called by some other name? You say the picture is overdrawn. Oh no, you all know men of that kind who are enjoying lucrative practices.

I would that it were overdrawn. I would that the time might come speedily when the requirement that men in order to receive a license to practice dentistry "shall be of a good moral character" might mean what it says, or else that the term "good moral character" might be interpreted from a higher standard.

In conclusion, then ,let us all do a little house cleaning. Let's aim a little higher. Let's give our patients better service, have more of the spirit of brotherly love among ourselves, and sterilize not only our instruments and our hands but our hearts also.—J. F. Cooke in *Tri-State Record.*

**Contour without Exaggeration.** In our struggle to attain in our dental operations the highest ideals, we can easily overdo the matter and produce results which in time prove to be detrimental.

This is particularly true when after extensive wedging or separating a number of contour fillings exaggerating the normal size of the filled teeth are inserted.

Many of us have seen mouths where extensive operations have been made with an eye to extension for prevention and found that each tooth was apparently elongated, an effect produced by the partial receding of the gums and straightening or stretching of the gum line.

These patients in many instances have suffered from acute sensitiveness of the necks of the teeth and the whole mouth has been put in a state of strain so that the normal articulation produces movements and gradual loosening of the teeth.

Different operators attain ideal contours in different ways. One operator whom I had the pleasure of watching filled out the whole interdental space with gold and then sawed out a wedge-shaped piece leaving the lower portion intact. This man produced the ideal contour, but in my estimation did it at great nerve cost to both himself and his patient.

An easier method, for which I claim no originality, enables one to attain the exact contour with the least expenditure of energy.

The cavity being prepared and the rubber dam adjusted, a matrix of such width and shape as one may need is cut from a sheet of thin steel.

Two orange wood sticks triangular in shape are sharpened to points and inserted back of the steel matrix, one being inserted from the palatal and one from the buccal side.

These two sticks pass each other and only sufficient force should be used in meeting them to press the matrix against the cervical margin and hold it there.

Great care must be exercised from beginning to end to thoroughly condense the gold, and when this filling is completed it needs no approximal disking by which many a good contour has been ruined.

The cervical edge can be trimmed with a sharp lance preferably and polished with strips, and the contour is as nature intended it.—Dr. O'Brian, *Items of Interest.*

**Immediate or Later Plate Insertion.**

How soon after the extraction of the anterior teeth should they be replaced with artificial ones? They may be replaced immediately by taking the impression before the teeth are extracted and cutting the plaster teeth from the cast; the denture can then be constructed the same as though the teeth had been previously extracted. In such cases the artificial denture should be placed as soon as the blood ceases to flow. This method is decidedly advantageous in those cases where there is considerable exposure of the gum in laughing, because a tooth can be used having a root extension, which will occupy the socket of the natural tooth, and will much retard the resorption of the gum tissue. In most cases it is best to wait until the swelling has subsided and the inflammation reduced before taking the impression. This will usually require from one to three weeks. It is a serious mistake for the patient to wait from six months to two years after the extraction of the six anterior teeth in either the upper or lower jaw before having the artificial ones inserted. There is but one thing in favor of waiting so long a time, and that is possibly saving the expense of the temporary denture; while there are several reasons against its advisability. We will consider only the most important one from an esthetic point of view, the wasting or shrinking of the muscles of expression. It will be almost impossible to get the patient and friends to acquiesce in the proper conformation of the artificial denture after such a lapse of time. If for no other reason than this, the artificial denture should be inserted in two to four weeks. The patient should be instructed that if the denture hurts, to persevere in wearing it, but to report at once for relief.

Usually in from three to six months the tissues will have resorbed so much that it will be best to "line up" the plate, although some cases will not require this attention. Usually a denture inserted after the teeth have been out one year will be quite durable.

There seems to be no well-defined idea as to how long a so-called permanent denture should be worn. It is the opinion of the writer that the best interests of the patient would be subserved if they did not wear any denture more than five or six years, unless upon examination it should be found there was perfect adaptation, and the base-plate was of a non-absorptive material.—G. H. Wilson, in *Dominion Journla.*

